# PARTNERS IN DEMENTIA – NORTHWEST: NEW APPROACHES IN MEDICAL STUDENT SERVICE LEARNING

**DOI:** 10.1093/geroni/igae098.1635

**Published:** 2024-12-31

**Authors:** Mengru Wang, Michael Rosenbloom, Marigrace Becker, Stephanie Adaniya

**Affiliations:** University of Washington, Seattle, Washington, United States; University of Washington, Seattle, Washington, United States; University of Washington, Seattle, Washington, United States; University of Washington School of Medicine, Seattle, Washington, United States

## Abstract

Partners in Dementia – Northwest is the first service-learning program at the University of Washington School of Medicine focused on older adults and persons living with memory loss, established in 2023. First year medical student volunteers, “mentees”, were paired with older adults living with memory loss, “mentors”, recruited from a memory disorders clinic based at the University of Washington. In its pilot year, 10 students were paired with 10 older adults and their caregivers, for bimonthly to monthly visits at the Memory Hub, a community space affiliated with the University that provides education, support, and wellness services. Students participate in mentor visits and group activities at the Memory Hub and accompany mentors to medical appointments to experience the health care system from the perspective of a person living with memory loss. The goals of the program are to provide medical students with early clinical exposure to memory disorders, reduce stigma surrounding aging and memory loss, and inspire students to pursue underserved specialties in dementia care. Pre-implementation surveys of students found that reasons for participation in the program included interest in learning more about the dementia disease process, building communication skills, recognizing signs of memory loss to enhance clinic skills, and learning how to maximize quality of life for persons with memory loss. Initial challenges identified by students include difficulties in engaging mentors when a caregiver is not present, navigating conversations, and transportation barriers.

